# Recommendations for Reducing the USE of Fish and Amphibians in Endocrine‐Disruption Testing of Biocides and Plant Protection Products in Europe

**DOI:** 10.1002/ieam.4156

**Published:** 2019-07-26

**Authors:** Laurent Lagadic, Katrin Bender, Natalie Burden, Edward R Salinas, Lennart Weltje

**Affiliations:** ^1^ Bayer AG, Crop Science Division, Environmental Safety, Ecotoxicology, Monheim am Rhein Germany; ^2^ NC3Rs London United Kingdom; ^3^ BASF SE, Agricultural Solutions ‐ Ecotoxicology Limburgerhof Germany

Criteria for determining the endocrine‐disrupting (ED) properties of biocide (European Union [EU] Regulation 2017/2100) and plant protection product (PPP) active substances (EU Regulation 2018/605) entered into force on 6 June and 10 November 2018, respectively. Accompanying the criteria is a guidance document (ECHA–EFSA–JRC 2018), which provides recommendations for evaluating the ED properties of chemicals for humans and nontarget organisms. It recommends selected tests to fulfill data sufficiency for either endocrine activity or adverse effects (defined by WHO/IPCS 2009 as a change in the morphology, physiology, growth, development, reproduction, or life span of an organism, system, population, or subpopulation that results in an impairment of functional capacity, an impairment of the capacity to compensate for additional stress, or an increase in susceptibility to other influences). A test indicating endocrine activity usually triggers a test for adversity. The present article focuses on data needs using fish and amphibians.

The suggested tests for mechanistic data sufficiency are the Amphibian Metamorphosis Assay (AMA; OECD TG 231, 2009) and Fish Short‐Term Reproduction Assay (FSTRA; OECD TG 229, 2012), whereas for adversity, the Larval Amphibian Growth and Development Assay (LAGDA; OECD TG 241, 2015b) and Medaka Extended One‐Generation Reproduction Test (MEOGRT; OECD TG 240, 2015a) are recommended. These tests require the use of many animals, are highly complex, and are infrequently conducted. Tests that are infrequently conducted (e.g., OECD TGs 240 and 241) are at greater risk of “failure” as a result of deviation from test guideline validity criteria (Salinas and Weltje 2018). A test that fails validity criteria usually requires repetition, doubling the number of animals used. Additionally, further animals are needed for range‐finding tests to set the appropriate concentration range for the definitive testing.

Due to the required test organism numbers, this testing conflicts with the aim to reduce vertebrate testing and to employ vertebrate testing only as a last resort (see Regulations EC 1107/2009 and EU 528/2012 on PPPs and biocides, respectively). In the present article, we estimate the number of fish and amphibians required for ED testing, based on registered PPPs and biocides in Europe.

In order to estimate animal numbers, we first estimated the number of biocides and pesticides that require an evaluation for ED properties. The PPP active substances, safeners, and synergists with status “approved” and “pending” were extracted from the European pesticide database (EC, 2016). Excluded were attractants, desiccants, elicitors, plant activators, pruning substances, repellents, soil amendments, and viral inoculates. This extraction and exclusion process resulted in 475 PPP active substances. Biocides with status “approved” or “approval in progress” were selected from the European Chemicals Agency database (ECHA 2019). Duplication of substances was corrected for, yielding 297 biocide active substances.

The number of animals required for each test was estimated on the basis of information provided in the test guidelines (Table 1). We assumed and advocate for the use of 1 control in case a solvent is used. For the FSTRA, the fish species that required the smallest sample size (i.e., zebrafish) was selected as a conservative approach. For each test, a range‐finding test was included with 3 test concentrations, a control and half the replicates of a definitive test. Also, a conservative failure rate (triggering test repetition) was estimated for each test based on the work of Burden et al. (2017), who surveyed test laboratories, and Salinas and Weltje (2018), who analyzed validation data.

**Table 1:** Estimated number of animals required for ED testing of registered pesticide and biocide active substances, based on range‐finding and definitive testing, and adjusted for failure rates

Test	Mechanistic assays		Adversity assays	
	FSTRA^a^ OECD TG 229	AMA OECD TG 231	MEOGRT OECD TG 240	LAGDA OECD TG 241
Number of animals in the range‐finding test	40	160	630	120
Number of animals in the definitive test^b^	96	320	1764	480
Failure rate for definitive test^c^	0.1	0.1	0.5	0.2
Total number of animals in test	128	512	3276	776
Number of animals for 475 pesticides	60 800	243 200	1 556 100	368 600
Total	304 000		1 924 700	
Number of animals for 297 biocides	38 016	152 064	972 972	230 472
Total	190 080		1 203 444	

AMA = Amphibian Metamorphosis Assay; ED = endocrine disrupting; FSTRA = Fish Short‐Term Reproduction Assay; LAGDA = Larval Amphibian Growth and Development Assay; MEOGRT = Medaka Extended One‐Generation Reproduction Test.

^a^ Numbers are provided for zebrafish; using fathead minnow or medaka as test species would increase the number of animals by 30%.

^b^ The number of animals for the definitive test was based on the minimum number of animals per replicate, the number of replicates per treatment and control, and the number of treatments (cf. the respective guidelines).

^c^ The failure rate describes the probability of test repetition due to inability to meet guideline validity criteria.

Our estimates indicate that, for mechanistic data sufficiency, 304 000 animals would be needed to fulfill the requirement of ECHA–EFSA–JRC (2018) for pesticides and 190 080 animals for biocides. The estimates for MEOGRT and LAGDA are higher (1.9 and 1.2 million animals for pesticides and biocides, respectively). The use of such high numbers of animals is not compatible with the desire to reduce animal testing in the European Union (EU).

We propose the following ways to reduce the test animal numbers, without compromising the knowledge needed to conduct a proper ecotoxicity evaluation for EDs:

- An obvious recommendation is to optimize test protocols to achieve lower failure rates (e.g., better validation, fewer and/or less ambitious validity criteria) and for authorities to require tests only following protocols with a low failure rate.
- Range‐finding tests can be omitted, provided that data are available from acute or chronic studies for setting appropriate concentrations in the definitive test. Although this is a reasonable assumption for fish, data are rarely available for amphibians. From acute studies, one‐tenth of the 96‐hr LC50 can be used as the highest test concentration in the FSTRA. However, a range‐finding test may be necessary if systemic toxicity or severe sublethal effects are observed near to one‐tenth of the LC50 (Wheeler et al. 2013). Although omitting the range‐finding test would result in a 31–36% reduction in the number of animals used in an FSTRA, this omission is rarely possible for the other tests.
- Make better use of embryo assays and mechanistic mammalian data. Embryo assays can provide in vivo mechanistic information. Fish embryo assays currently undergoing Organisation for Economic Co‐operation and Development (OECD) validation can detect substances acting through the Estrogen, Androgen or Steroidogenesis (EAS) modalities (detection of Endocrine Active Substances acting through estrogen receptors, using Zebrafish embrYos [EASZY]; Rapid Estrogenic Activity Test In Vivo [REACTIV]; or Rapid Androgen Disruption Adverse Outcome Reporter Assay [RADAR]). For detecting thyroid activity, an amphibian embryo assay, Xenopus Eleutheroembryonic Thyroid Assay (XETA), has been adopted by the OECD. These assays are considered as “nonanimal” tests within the EU because they use embryos at a development stage where independent feeding has not yet started, whereas both the United Kingdom Animals (Scientific Procedures) Act 1986 and the Directive 2010/63/EU on the protection of animals used for scientific purposes apply to only independently feeding larval forms. The embryo assays used for investigating endocrine mechanisms therefore appear as an ethical alternative to tests conducted with older larval stages, juveniles, or adults (Halder et al. 2010). Incorporating embryo assays into testing strategies for ED testing is consistent with EU legislative animal welfare objectives, if the FSTRA or AMA were conducted only when embryo assays indicate endocrine activity.
- Avoiding duplication of mechanistic studies across vertebrates due to the high level of conservation of the endocrine system and receptor homology, as well as the key enzymes involved, extrapolation of qualitative screening‐level information among vertebrates is warranted. There is a high concordance between the results of amphibian or fish and rat assays for substances that interact with Estrogen, Androgen, Thyroid, Steroidogenesis (EATS) modalities (Ankley and Gray 2013; Pickford 2010). This has been confirmed by comparing protein sequence and/or structural information across species at the level of the primary amino acid sequence and functional domains (LaLone et al. 2013). Therefore, it is questionable whether additional mechanistic studies for aquatic vertebrates are needed when endocrine activity has been investigated sufficiently in mammalian assays.

Overall, the evaluation of ED properties for pesticides and biocides will result in a vast increase of the number of animals used in testing. However, utilizing cross‐species extrapolation considerably reduces animal use, if no further tests on fish and amphibians are required when endocrine activity is sufficiently investigated in mammalian assays. In vivo embryo assays offer an ethical alternative to corroborate mammalian results and robustly address environmental protection goals for aquatic vertebrates.
